# Recurrent hiatal hernia causing small bowel obstruction secondary to herniation into right hemithorax following prior esophagectomy: a case report

**DOI:** 10.1093/jscr/rjaf381

**Published:** 2025-06-30

**Authors:** Peculiar A Ihunwo, Sarah McIntyre, John K Saunders

**Affiliations:** Department of Surgery, NYC Health + Hospitals/Bellevue, 462 1st Ave., New York, NY 10016, United States; Department of Surgery, NYU Langone Health, 550 1st Ave., New York, NY 10016, United States; Department of Surgical Oncology, Roswell Park Comprehensive Cancer Center, 699 Elm St., Buffalo, NY 14263, United States; Department of Surgery, NYC Health + Hospitals/Bellevue, 462 1st Ave., New York, NY 10016, United States; Department of Surgery, NYU Langone Health, 550 1st Ave., New York, NY 10016, United States; Department of Surgery, VA NY Harbor Healthcare System, 423 E 23rd St., New York, NY 10010, United States

**Keywords:** hiatal hernia, small bowel herniation, esophageal adenocarcinoma, laparoscopic surgery, small bowel resection

## Abstract

Hiatal hernias occur when abdominal contents, typically the stomach, herniate through the esophageal hiatus into the mediastinum, causing symptoms such as reflux, chest pain, and dysphagia. Surgical repair aims to restore anatomy and prevent complications such as obstruction or strangulation. While sliding hernias are more common, paraesophageal hernias more frequently necessitate surgery. A case of small bowel herniation into the right hemithorax through a recurrent hiatal hernia in a 77-year-old male with a history of reflux and stage II esophageal adenocarcinoma treated with neoadjuvant chemoradiation, immunotherapy, and esophagectomy. He presented with abdominal pain and dyspnea. Computed tomography imaging revealed small bowel obstruction due to herniation into the thoracic cavity. Emergent intervention included laparoscopic hiatal hernia repair and open small bowel resection. The patient’s postoperative course was uneventful, and follow-up imaging at 3 months showed no recurrence. This case underscores the importance of timely surgical intervention in managing complex recurrent paraesophageal hernias.

## Introduction

Hiatal hernias occur when abdominal organs, most commonly the stomach, protrude into the thoracic cavity through the esophageal hiatus. While sliding hiatal hernias are common and often asymptomatic, paraesophageal hernias, particularly recurrent ones, present unique challenges and complications, including obstruction, strangulation, and ischemia [1]. The management of recurrent hiatal hernias following esophagectomy is particularly complex, requiring a multidisciplinary approach to minimize the risk of complications and improve patient outcomes [2]. This case report highlights a patient with a history of esophagectomy who presented with a recurrent hiatal hernia causing small bowel obstruction. The surgical strategy employed, incorporating laparoscopic repair with pledget reinforcement, underscores the importance of precise operative techniques to optimize recovery and reduce the risk of recurrence. This approach has been documented in the literature to enhance the durability of the repair and minimize postoperative complications [3, 4].

## Case presentation

### Patient information

A 77-year-old male with a significant medical history including bipolar disorder, basal and squamous cell carcinoma status post excision, hiatal hernia, reflux, coronary artery disease, remote alcohol use disorder, former smoker, and a recent diagnosis of stage II mid-distal esophageal adenocarcinoma treated with neoadjuvant chemotherapy/radiotherapy, immunotherapy, and esophagectomy. The patient also had a history of a hiatal hernia repaired with mesh. The patient presented to the emergency department (ED) with 2 days of abdominal pain and emesis.

### Clinical findings and pre-op assessment

**Figure 1:**
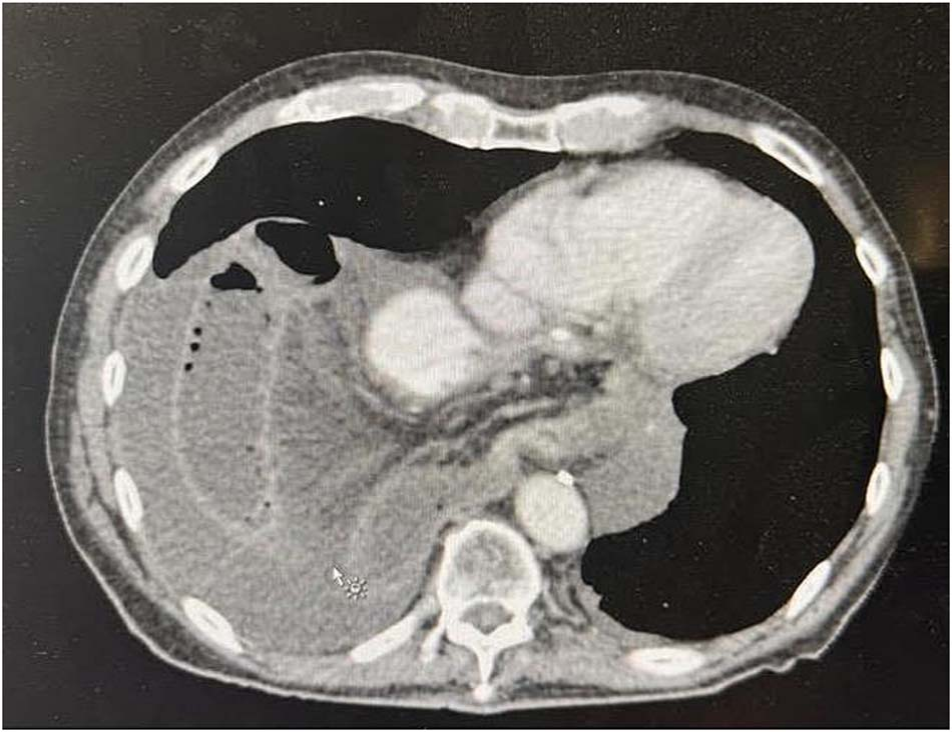
Preoperative CT image showing recurrent hiatal hernia with dilated small bowel in the right hemithorax, mesenteric edema, and surrounding fluid.

**Figure 2:**
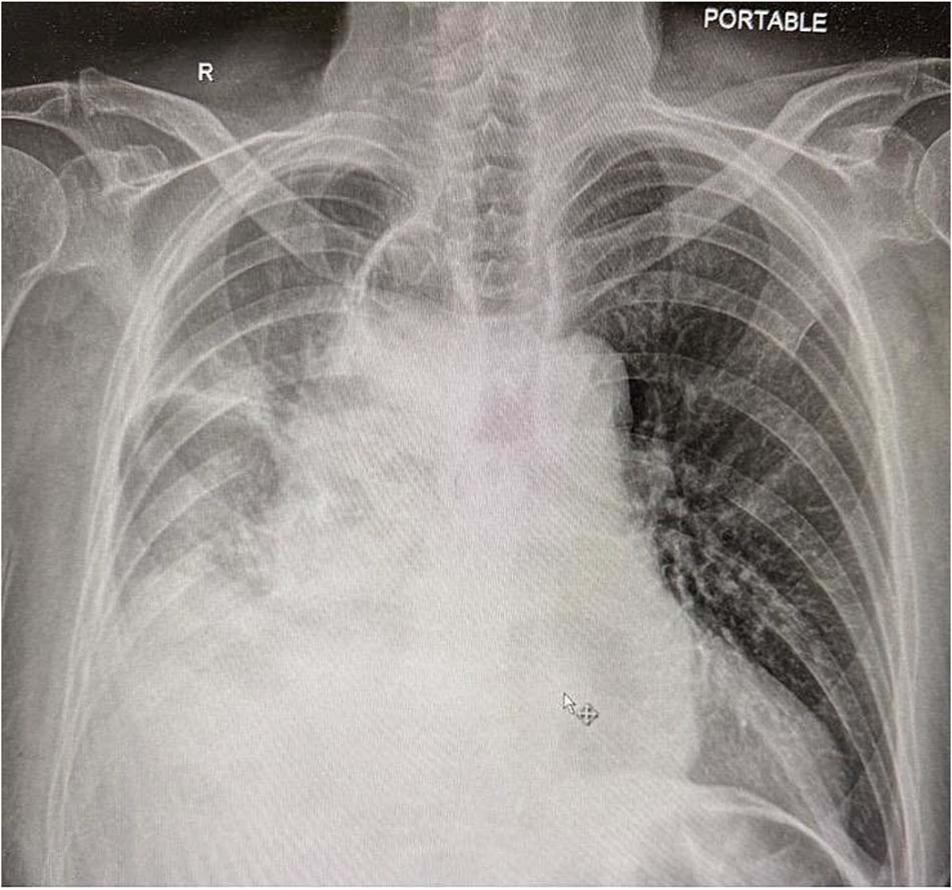
Chest X-ray showing compressed lung and fluid due to herniated small bowel.

Upon arrival at the ED, the patient was afebrile and hemodynamically stable. Initial labs showed WBC 10.19, H/H 13.2/41.3, platelets 419, Na 136, K 5.3, creatinine 1.0. Portable X-ray and computed tomography (CT) imaging revealed small bowel obstruction secondary to herniation of the small bowel into the right hemithorax through a recurrent hiatal hernia (Figs 1 and 2).

## Surgical procedure

**Procedure:**

(1) Laparoscopic exploration
(2) Hiatal hernia repair
(3) Small bowel resection via mini-laparotomy

**Preoperative diagnosis:** Small bowel obstruction

### Procedure in detail

The patient was positioned split-leg for optimal access. The abdomen was accessed using a Veress needle and optiview. On inspection, dilated small bowel loops and a hiatal hernia were identified. Additional 5 mm ports were placed to triangulate to the hiatus. A Thompson liver retractor was placed to improve exposure.

The gastric conduit appeared normal. Herniated bowel loops were reduced by incising the left crus and removing part of the mesh. A primary repair with pledget reinforcement was then performed laparoscopically. After confirming hemostasis, the ischemic portion of the bowel was resected through a mini laparotomy, and a stapled anastomosis was performed. The mesenteric defect was closed with Vicryl, and the fascia and skin were sutured with PDS and nylon, respectively. *A video of the procedure demonstrating laparoscopic reduction, pledget-reinforced repair, and small bowel resection is available as supplementary material (Video S1).*

### Post-op follow-up and management

**Figure 3:**
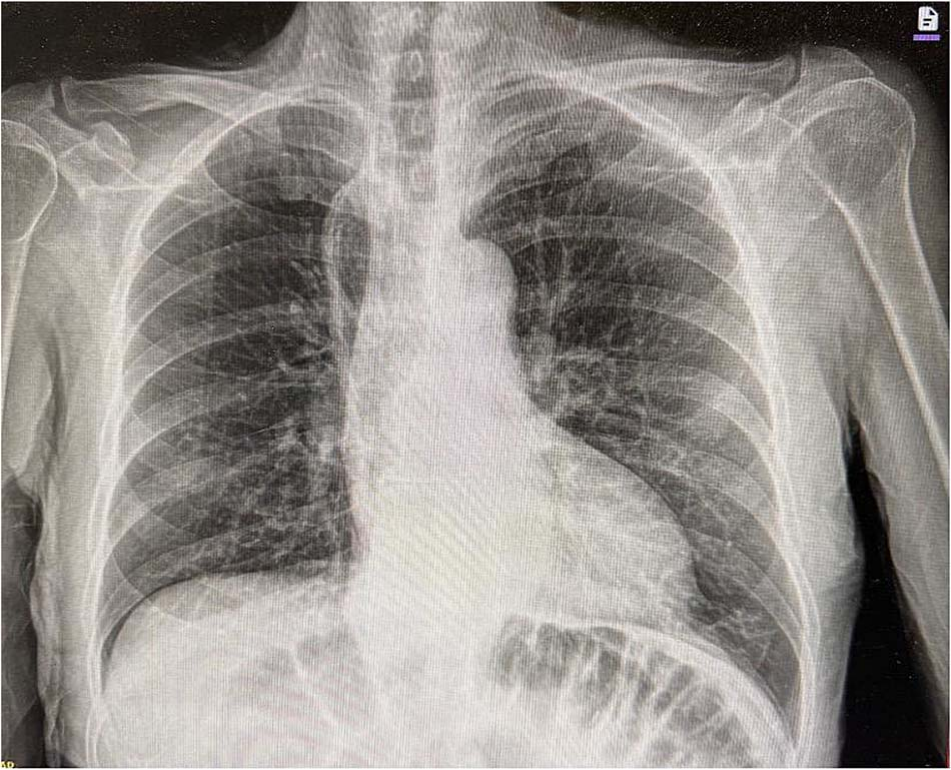
Postoperative chest X-ray imaging.

The patient’s postoperative course was relatively uncomplicated. He returned to bowel function on postoperative day 2. A superficial surgical site infection was managed with local wound care. Imaging obtained 2 months after surgery demonstrated no recurrence of the hernia (Fig. 3).

## Discussion

This case highlights the complexity of recurrent hiatal hernias with small bowel herniation. Similar cases emphasize the importance of timely surgical intervention to prevent complications. Previous studies have shown that recurrent hiatal hernias, especially those involving small bowel herniation, require prompt and effective surgical management to prevent morbidity and mortality.

Effective multidisciplinary collaboration was crucial. Thoracic surgery was consulted during the operation to discuss the best repair for this patient. The use of pledget reinforcement for repair and the decision to resect the dusky bowel were pivotal. The necessity of careful monitoring and timely surgical intervention in recurrent hiatal hernia cases was evident. Studies suggest early surgical intervention in recurrent hernia cases can significantly improve patient outcomes.

Hiatal hernia recurrence is a significant issue following repair surgery, with rates influenced by the chosen surgical technique, patient characteristics, and mesh reinforcement. Recurrence rates for laparoscopic hiatal hernia repairs without mesh have been reported to range from 15% to 42%, largely due to the difficulty in achieving tension-free closure with minimally invasive techniques [1]. The use of pledget reinforcement for repair in such cases has been documented to reduce recurrence rates [4]. In contrast, open procedures tend to provide better structural support, although they come with increased recovery times and potential for greater morbidity [5].

Studies have investigated the effectiveness of mesh reinforcement in reducing recurrence rates. Reports in the *Journal of Surgical Case Reports* show that mesh placement can offer additional support to the crural closure, potentially lowering recurrence risk in the early postoperative period [3]. However, long-term data remain inconclusive. Randomized controlled trials demonstrate conflicting results, with some indicating no significant difference in recurrence rates when comparing mesh to suture-only repairs. This may be attributed to complications like mesh erosion and migration, which sometimes negate its benefits [4]

In patients who undergo esophagectomy, particularly minimally invasive esophagectomies (MIE), the incidence of diaphragmatic hernias, including para-conduit hernias, can be as high as 20%, compared to the 5%–10% seen with open procedures. This increase is likely due to the extensive mediastinal dissection performed in MIE, which can weaken the hiatus and predispose it to herniation [6]. Literature in the *Oxford Medical Case Reports* supports this, emphasizing the need for careful planning and technique selection during esophagectomy to reduce hernia risks.

Patient-specific factors such as body mass index (BMI) also play a crucial role in recurrence risks. Some research suggests that a BMI greater than 25 kg/m^2^ provides a protective effect by stabilizing the intra-abdominal contents, reducing visceral mobility. However, other studies indicate that higher intra-abdominal pressure in obese patients might actually increase recurrence risk [4]. Pre-existing hiatal hernias are also a known risk factor for postoperative incarceration and recurrence, highlighting the necessity for a personalized surgical approach.

Neoadjuvant therapy, including chemotherapy and radiation, is another critical aspect influencing recurrence rates. Some studies suggest that these treatments delay healing and increase the risk of recurrence, while others propose that the fibrosis resulting from radiation may help stabilize the hiatus, thus reducing the likelihood of herniation [2]. This duality underscores the complexity of neoadjuvant effects and necessitates careful consideration in surgical planning.

Ultimately, managing hiatal hernia recurrence, particularly after esophagectomy, requires an individualized and multidisciplinary approach. Decisions regarding mesh use should be tailored based on the patient’s risk profile, weighing the benefits of structural support against the potential for complications. A high degree of clinical suspicion, especially in high-risk patients, remains essential for early detection and management of recurrence. Effective collaboration among surgeons, oncologists, and gastroenterologists is key to optimizing patient outcomes and reducing recurrence risks.

## Conclusion

This case demonstrates the successful minimally invasive management of a complex recurrent hiatal hernia with small bowel herniation, emphasizing the importance of multidisciplinary care, timely intervention and meticulous surgical technique. Clinicians should have a high index of suspicion for hiatal hernia recurrence, especially in high risk patients.

## Supplementary Material

Hiatal_Hernia_Case_Report_Video_Updated_300mb_rjaf381
